# Stigmatization Is Associated With Increased PTSD Risk After Traumatic Stress and Diminished Likelihood of Spontaneous Remission–A Study With East-African Conflict Survivors

**DOI:** 10.3389/fpsyt.2018.00423

**Published:** 2018-10-10

**Authors:** Anna Schneider, Daniela Conrad, Anett Pfeiffer, Thomas Elbert, Iris-Tatjana Kolassa, Sarah Wilker

**Affiliations:** ^1^Department of Clinical & Biological Psychology, Institute of Psychology and Education, Ulm University, Ulm, Germany; ^2^Vivo International e.V., Konstanz, Germany; ^3^Department of Psychology, University of Konstanz, Konstanz, Germany

**Keywords:** stigmatization, discrimination, mental health, posttraumatic stress disorder (PTSD), narrative exposure therapy, post-conflict population, spontaneous remission, treatment outcome

## Abstract

Studies in conflict population have repeatedly documented that the number of traumatic event types experienced (trauma load) increases the risk to develop posttraumatic stress disorder (PTSD) in a dose-dependent manner. Misconceptions about survivors' experiences and actions during the war, as well as mental health symptoms frequently lead to stigmatization by their own families and the community, which might render them even more vulnerable for PTSD development and prevent successful recovery. We therefore investigated whether stigmatization affects trauma-related psychopathology beyond the well-known effect of trauma load. The study sample comprised *N* = 1131 survivors of the rebel war led by the Lord's Resistance Army (LRA) in Northern Uganda, including a large proportion of formerly abducted individuals and child soldiers. We investigated how the experience of stigmatization affects PTSD risk and the likelihood of spontaneous remission, taking trauma load into account. Further, the association of stigmatization with treatment outcome was determined in a subsample of *N* = 284 individuals with PTSD who received trauma-focused psychotherapy. More than one third of the total sample, and almost two-thirds of the therapy subsample, reported experiences of stigmatization. The main reasons for stigmatization were related to an association with a rebel group (e.g., being called a rebel), followed by mental health problems/PTSD symptoms and HIV/AIDS. Stigmatization was strongly associated with a higher prevalence of lifetime and current PTSD, a diminished probability of spontaneous remission and higher PTSD symptoms before and after trauma-focused psychotherapy, beyond the effect of trauma load. In sum, our results support the assumption that stigmatization aggravates trauma-related psychopathology and impede symptom improvement. In post-conflict regions, community and family interventions which aim at reducing stigmatization and discrimination might therefore complement individual psychotherapy in order to allow survivors to recover and reintegrate into society.

## Introduction

Stigmatization is usually caused by misconceptions and social disapproval based on different beliefs or perceptions, which lead to negative stereotyping and labeling of an individual, and can be followed by social exclusion and unjust treatment (1–3). A meta-analysis, mainly based on empirical studies conducted in Western countries, found stigmatization to be associated with poorer mental health and reported different reasons for the experience of stigmatization, e.g., symptoms of mental health problems, gender, HIV/AIDS, and sexual abuse during childhood (4). In accordance with that, Betancourt et al. (5) showed that the perceived stigmatization of former child soldiers from Sierra Leone correlated positively with levels of anxiety, hostility and depression. Further studies conducted in post-conflict settings associated stigmatization with decreases in prosocial behavior in children, as well as internalizing and externalizing problems, leading to even more stigmatization (6–8).

So far, only a small body of research investigated stigmatization as an essential risk factor for the development of mental diseases in post-conflict societies. Different studies conducted in post-conflict areas agree on a particularly high burden of stigmatization, rejection and exclusion of girls and women who experienced sexual violence and returned from captivity with children conceived from rape (9–11). Further, Betancourt et al. (5) showed that 71% of former child soldiers from Sierra Leone reported stigmatization. The main reasons were *being a former child soldier, female gender* and the *low financial status* of the family. It can be assumed that continuing stigmatization and discriminating actions have a strong negative effect on everyday life, leading to low self-esteem, long-lasting negative psychological effects and finally, preventing a successful re-integration into society (5, 12, 13).

To this day limited research has been conducted investigating the direct impact of stigmatization on PTSD risk in a post-conflict context. Studies conducted with survivors of sexual assault showed that the reaction of society, including stigmatizing responses, was highly predictive of PTSD symptom severity (10, 14, 15). Similar findings were shown by a study examining HIV positive women, showing that the severity of stigmatization was the strongest predictor for PTSD risk besides physical HIV symptoms and negative life change (16).

Research in post-conflict settings has repeatedly shown that the experienced amount of different types of traumatic events (trauma load) increases PTSD risk in a dose-dependent manner and leads to prevalence rates of up to 100% at extreme levels of trauma exposure (17–21). Furthermore, a higher trauma load decreases the likelihood of spontaneous remission (22) and results in the persistence of higher PTSD symptoms even after effective psychotherapy (Schneider et al., submitted). Nevertheless, it remains unclear how much variability of PTSD risk, spontaneous remission and therapy outcome, can be explained through stigmatization beyond the robust effect of trauma load. The investigation of the effects of stigmatization on trauma-related psychopathology is particularly interesting since, unlike trauma load, stigmatization can be addressed through interventions.

We investigated the effects of stigmatization in Northern Uganda, a region that was severely affected by the war between the rebel group “Lord's Resistance Army” (LRA) and the Ugandan governmental soldiers for almost 20 years. Between 1986 and 2006 the LRA forcibly recruited approximately 60,000–80,000 people, most of them children (23, 24). Individuals abducted by the LRA were forced to commit atrocities, e.g. murder friends and families. Abducted girls were given to commanders as “wives,” leading to a huge number of rapes and unwanted pregnancies (25). In a population-based study on the post-conflict population of Northern Uganda, 98% of the participants reported at least one traumatic event, and 25% of former child soldiers fulfilled the diagnosis of PTSD at the time of the assessment (18).

Even though the Ugandan government granted amnesty for the returnees (26), many of them still face stigmatization in their day to day lives. Re-integration into civil society appears to be difficult, as returnees are frequently confronted with different social, economic, medical and psychological problems (9). As a consequence of the time spent in captivity or of the experienced atrocities by the LRA, many survivors suffer from medical issues, like HIV, present with mental health impairments, e.g., PTSD, depression, suicidality, and report to be stigmatized. In studies with individuals returning from the LRA (9, 23), every tenth participant reported rejection by his or her family and community. Of the interviewed women, 39% were called names and 35% had the feeling that their communities were afraid of them. Another five percent even reported the experience of physical violence by their relatives (9).

The aims of the present study were two-fold: First, we intended to investigate the prevalence of stigmatization in an exceptionally large cohort of Northern Ugandan rebel war survivors and assess the main reasons for stigmatization. Second, we wanted to determine the impact of the experienced stigmatization on PTSD risk taking trauma load into account, and unravel its impact on the likelihood of spontaneous remission and therapy success.

## Materials and methods

### Sample

The study was part of a larger project investigating gene × environment interactions in PTSD etiology and treatment among survivors of the war between the rebel group LRA and the Ugandan governmental troops. On the one hand measures for the accurate assessment of the environmental factor trauma load were analyzed (27, 28), on the other hand genetic factors, in particular memory-related genes, were investigated in previous studies (29, 30). The data was collected in the former Internal Displaced People (IDP) camps Anaka, Pabbo and Koch Goma, and in villages of Gulu and Nwoya district, Northern Uganda. In total, 1813 individuals were interviewed and provided written informed consent prior to study participation. We excluded individuals with missing information on the experience of stigmatization or discrimination (*N* = 638), signs of current alcohol abuse (*N* = 11) and missing data regarding current and lifetime PTSD diagnostic status from the study (*N* = 33). Thus, statistical analyses on PTSD risk were based on a sample of *N* = 1131 (627 females, *M*_age_ = 32.80, *SD*_age =_ 10.38, age range: 18–80 years). For investigations on the effect of stigmatization on treatment outcome a subsample of this cohort (*N* = 317) who received psychotherapy was used and will hereafter be referred to as *therapy sample*. All individuals in the therapy sample fulfilled the criteria for a current PTSD diagnosis according to DSM-IV-TR (31) with a minimum symptom score of 10 at the time of the first assessment and were offered treatment with Narrative Exposure Therapy [NET; according to the manual (32)]. Exclusion criteria were the use of psychotropic medication or former trauma-focused therapy, current signs of alcohol or substance addiction, presence of psychotic symptoms and age under 18 years. Since four individuals refused to participate in NET and 29 dropped out or were excluded from the study (see Supplementary Material for more information on drop-out and exclusion reasons) the sample used for statistical analyses on treatment outcome comprised *N* = 284 individuals (160 females, M_age_ = 32.49, SD_age_ = 9.33, age range: 18–62 years).

### Materials and study procedure

The Institutional Review Board of Gulu University, the Lacor Hospital Institutional Research Ethics Committee, the Ugandan National Council for Science and Technology (UNCST), Uganda, and the ethics committee of the German Psychological Society (Deutsche Gesellschaft für Psychologie, DGPs), approved the study procedures. Local counselors, who were intensively trained on the concepts of mental health disorders, trauma and PTSD, counseling skills, and quantitative data collection, performed the interviews under close supervision of experienced psychologists. All diagnostic instruments were translated into Luo, the local language of Northern Uganda, following a procedure of translation, blind back-translation and independent review by trained interpreters to avoid any misinterpretation.

#### Demographics and stigmatization

Besides demographic information, participants were asked whether they perceive stigmatization or discrimination (Do you feel people are stigmatizing or discriminating you [e.g., are bullying you, calling you names, laugh at you, act surprised or startled when they see you, or don't know what to say to you?]). Even though the item assessed both stigmatization and resulting discriminating actions, we will in the following only use the term “stigmatization” for a better readability. If answered with “yes,” participants were furthermore asked to describe the reason(s) for stigmatization. For a descriptive overview of these reasons, the qualitative answers were assigned to the following 14 categories by two independent raters: LRA-related, HIV/AIDS, physical injury/disease (other than HIV/AIDS), mental health problems/PTSD symptoms, family-related problems, land issues, financial problems, different ethnicity, widowed, orphan, low education, political reasons, unknown and others. In case of a mismatch between the two raters, an independent third rater made a final decision. In the subsequent analyses, the impact of stigmatization on mental health outcomes was analyzed irrespective of the reason(s) for stigmatization (cf. Table 1).

**Table 1:** Reasons for stigmatization in total sample and therapy sample.

**Sample**	**Reasons for stigmatization (sorted by frequency in decreasing order)**	***N***
Stigmatized individuals in total sample (*N* = 352; 31.12%)	LRA-related	216
	HIV/Aids	37
	Mental health problems/PTSD symptoms	31
	Family-related problems	26
	Others	24
	Physical injury/disease (other than HIV/Aids)	12
	Unknown	9
	Orphan	8
	Land-issues	7
	Different ethnicity	5
	Widowed	3
	Financial problems	3
	Low education	3
	Political reasons	1
Stigmatized individuals in therapy sample (*N* = 172; 57.53%)	LRA-related	134
	Mental health problems/PTSD symptoms	19
	HIV/Aids	9
	Family-related problems	4
	Land-issues	4
	Others	4
	Unknown	3
	Financial problems	3
	Different ethnicity	3
	Widowed	2
	Orphan	2
	Low education	1
	Physical injury/disease (other than HIV/Aids)	1
	Political reasons	0

*PTSD, Posttraumatic Stress Disorder. Multiple answers were possible, therefore no percentages are reported. Reasons for stigmatization were sorted by frequency in decreasing order*.

#### Trauma event types

Trauma exposure was assessed with a 62-item event list already used and validated in previous studies (28–30), which included events related to war and violence in general, the LRA war in particular, natural disasters, domestic violence and other traumatic events (e.g., life-threatening illness or accidents). Participants should indicate whether they ever experienced the described event or not. Afterwards, the sum score of the different traumatic event types experienced was calculated as a precise and the most economic measure of trauma exposure (27, 28).

#### Posttraumatic stress disorder (PTSD)

The diagnosis of current and lifetime PTSD according to DSM-IV-TR (31) was assessed with the Posttraumatic Stress Diagnostic Scale [PDS; (33)], which was applied as a diagnostic interview. In addition to exposure to a traumatic event, participants had to fulfill one symptom in the intrusion cluster, three symptoms in the avoidance cluster and one symptom in the hyperarousal cluster. Furthermore, a functionality impairment as well as a symptom duration of at least one month was required to fulfill the diagnosis of PTSD.

The reliability and validity of the translated PDS has been previously assured for this context (34). An individual was classified as spontaneously remitted when he or she presented with a PTSD diagnosis in the past, but did not fulfill the symptom requirements for a current PTSD diagnosis any more at the time of the interview.

#### Narrative exposure therapy (NET)

NET is an exposure-based short-term therapy that has been developed particularly for survivors of multiple traumatic experiences suffering from PTSD (32). A common problem among PTSD patients constitutes their inability to allocate trauma-related cognitions and emotions to the past, resulting in the unwanted re-experience of traumatic memories with here and now quality, e.g., in the form of flashbacks or nightmares. During NET, the therapist assists the patients in re-integrating the defragmented memories of the traumatic experiences into a coherent and chronological narrative. By means of imaginative exposure, the client can thus locate the origins and cues of fearful emotions and learns to discriminate actual threats from traumatic memories of the past, which finally leads to symptom reduction (32). The effectiveness of NET for the treatment of PTSD in resource-poor as well as high-income countries has been shown in multiple studies [for reviews see (35, 36)]. Furthermore, NET has been successfully disseminated to local counselors in different conflict regions including Northern Uganda, who provided treatment with similar outcomes as expert psychologists (37–39).

In this study, well trained local counselors conducted the treatments under close supervision of expert psychologists. Treatment adhesion was monitored by weekly supervision meetings, intense case discussions, and a close review of the therapy session protocols. Participants received on average 12 sessions of NET that lasted 90–120 min and generally took place twice a week. Diagnostic assessments were carried out before treatment (t_1_), and four (t_2_), and 10 months (t_3_) after the end of treatment. The timing of the follow-up assessments was chosen since NET, which aims at a chronological reconstruction of autobiographical memory, initializes a process of recovery which continues over time. Confirming this, evidence from Northern Uganda shows that NET treatment effects increase over time (39).

### Data analysis

All statistical analyses were performed with R version 3.4.1 (40). The alpha level for all analyses was 0.05.

#### Stigmatization and the likelihood of PTSD and spontaneous remission

To investigate whether the prevalence for lifetime or current PTSD is higher among stigmatized than non-stigmatized individuals, *Chi*^2^-tests were applied. Furthermore, we hypothesized that stigmatization predicts lifetime and current PTSD risk beyond the influence of trauma load and may even intensify the cumulative effect of trauma load on PTSD risk. Both diagnostic variables were binary coded into 0 (no diagnosis) and 1 (diagnosis). Logistic regression models of increasing complexity were compared based on Akaike's information criteria (AIC) (41) and included trauma load, stigmatization and their interaction, to test whether the effect of stigmatization remains stable across all levels of trauma load. Further, sex and age were included as covariates. The assumptions of logistic regressions [no outliers, no multicollinearity, linearity of the logit; (42, 43)] were tested and did not reveal significant multicollinearity (variance inflation factor ≤ 1.1 in all analyses) or non-linearity of the logit. Nevertheless, we detected a few outliers. However, since extreme values of trauma load can be expected in this sample and represent valid data points, and since logistic regression analysis is known to be robust to outliers in large samples, outliers were not removed from the analyses (44, 45). Similar to the procedures for current and lifetime PTSD, *Chi*^2^-tests were used to investigate stigmatization-dependent differences in the prevalence for spontaneous remission. Further, logistic regression models were calculated to unravel the effect of stigmatization on the likelihood of spontaneous remission using the same model selection procedure as described for lifetime and current PTSD. By exponentiating the regression coefficients of logistic regression models, the odds ratio (OR) can be obtained as a measure of effect size. The OR represents the change in the relative probability of the dependent variable being true, e.g., the patient has a PTSD diagnosis, if the independent variable increases by one unit. Hence, an OR > 1 represents an increase and an OR < 1 a decrease in probability. An OR of 1 means no change in probability (46). To evaluate the statistical significance of stigmatization, trauma load and the covariates included in the models for binary outcome variables, we calculated likelihood ratio (LR) tests (47).

#### Stigmatization and therapy outcome

Finally, linear mixed effect models were used to investigate whether stigmatization similarly influences PTSD treatment response, i.e., changes in current PTSD symptom severity over time [R package “nlme” version 3.1.120; (48)]. Therefore, models were compared with the AIC and included the PDS sum score as outcome variable, time as a within-subject fixed factor, stigmatization as a between-subject fixed factor and their interaction. The predictor variable time was factorized to be able to test for non-linear symptom reduction. Additionally, the covariates trauma load, sex and age were included as additional fixed factors and participants were modeled as a random effect, with random intercepts for each participant. Due to non-normally distributed model residuals, statistical significance was evaluated by means of permutation tests using 1,000 random permutations and empirical *p*-values (*p*_emp_) are reported. Cohen's d is reported for the treatment effect size. In addition, *Chi*^2^-tests were calculated to investigate whether stigmatized compared to non-stigmatized individuals have a higher risk to still fulfill the diagnostic criteria for current PTSD four and 10 months after the end of therapy.

Finally, it has to be noted that although all study participants survived the same conflict, the time of trauma exposure varied strongly between the participants, as the LRA conflict lasted two decades. To test for a potential influence of the time interval between the worst traumatic experience and the interview on our results, we repeated all our analyses including the variable “time since worst traumatic event”.

## Results

### Descriptive statistics

Our total sample comprised *N* = 1131 individuals with complete information on the experience of stigmatization (627 females, M_age_ = 32.80, SD_age_ = 10.38, age range: 18–80 years). Of those, *N* = 352 (31.12%) participants reported that they felt stigmatized, while this was not the case in the remaining sample (*N* = 779, 68.88%). The major reason for stigmatization (*N* = 216) concerned the individual's abduction by and experiences within the LRA (e.g., people say that I am a rebel, that I was in the bush with the LRA, that I am a former abductee or a returnee and that I killed other people). The second most prevalent reason for stigmatization was HIV/AIDS (*N* = 37), followed by mental health problems/PTSD symptoms (*N* = 31). More than half of the individuals in the therapy subsample (*N* = 284) perceived stigmatization (*N* = 166; 58.45%). Similar to the total sample, a history with the LRA was the main reason for stigmatization (*N* = 134), followed by mental health problems/PTSD symptoms (*N* = 19) and HIV/AIDS (*N* = 9). For an overview of the reasons for stigmatization in both samples see Table 1.

### Stigmatization and the risk for PTSD

First, we investigated whether the prevalence for lifetime and current PTSD was higher among stigmatized than non-stigmatized individuals. For the exact numbers of healthy individuals and those with lifetime and current PTSD diagnosis in each group, as well as for a demographic and clinical data comparison, see Table 2. *Chi*^2^-tests indicated a significantly higher prevalence of lifetime [χ^2^(1) = 63.68, *p* < 0.001] and current PTSD [χ^2^(1) = 191.91, *p* < 0.001] among stigmatized compared to non-stigmatized individuals.

**Table 2:** Comparison of demographic and clinical data of stigmatized and non-stigmatized individuals in the total sample.

**Variable**	**Stigmatized group (*N* = 352)**	**Non-stigmatized group (*N* = 779)**	**Statistics**
*N* female (%)	208 (59.09)	419 (53.79)	Fisher's exact test: *p* = 0.106^a^
Age	Mdn = 31, IQR = 13	Mdn = 31, IQR = 17	*U* = 14,1693, *p* = 0.348^b^
Trauma load	Mdn = 33.5; IQR = 12	Mdn = 25; IQR = 13	*U* = 73,621, *p* < 0.001^b^
*N* lifetime PTSD diagnosis (%)	316 (89.77)	523 (67.14)	χ^2^(1) = 63.68, *p* < 0.001^c^
*N* current PTSD diagnosis (%)	218 (61.93)	155 (19.90)	χ^2^(1) = 191.91, *p* < 0.001^c^
PDS sum score t1	Mdn = 13, IQR = 13	Mdn = 2, IQR = 7	*U* = 71,878, *p* < 0.001^b^
*N* spontaneous remission (%)	98 (31.01^1^)	368 (70.36^2^)	χ^2^(1) = 121.94, *p* < 0.001^c^

*Mdn, Median; IQR, Interquartile range; PTSD, Posttraumatic Stress Disorder; PDS, Posttraumatic Stress Diagnostic Scale*.

a *Fisher's exact test for count data*.

b *Mann-Whitney U-test for continuous data, as model residuals were not normally distributed*.

c *Chi^2^-test for categorical data*.

1 *Based on a total of N = 316 stigmatized individuals showing a lifetime PTSD*.

2 *Based on a total of N = 523 non-stigmatized individuals showing a lifetime PTSD*.

**Figure 1:**
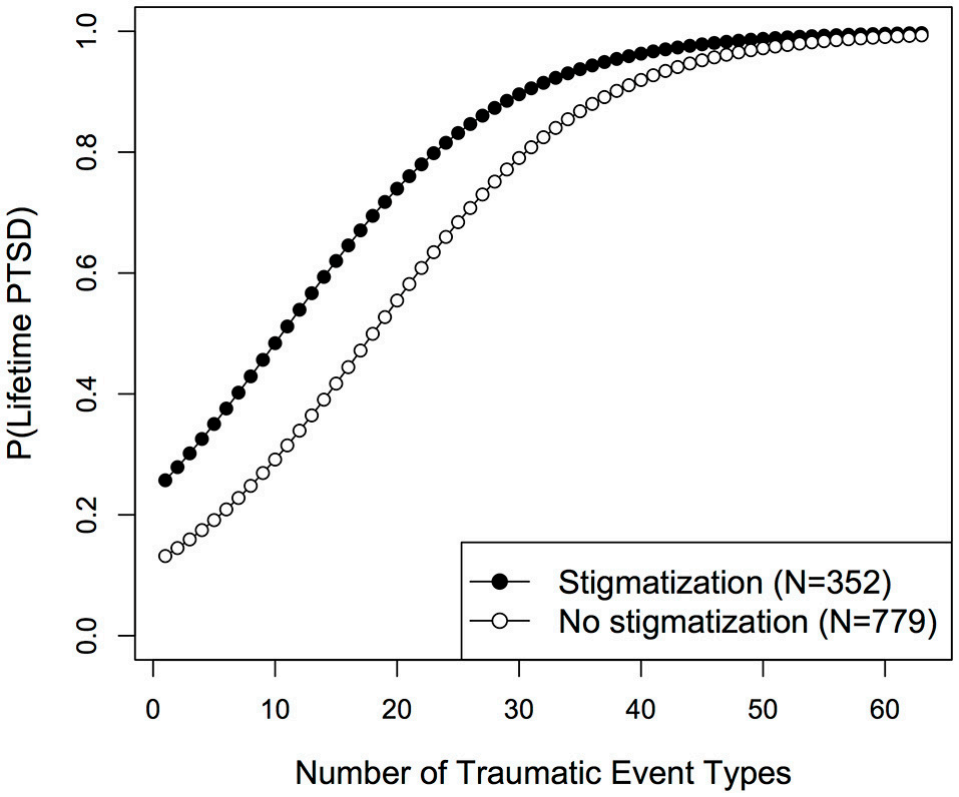
Fitted probabilities for lifetime PTSD development among stigmatized and non-stigmatized individuals.

**Figure 2:**
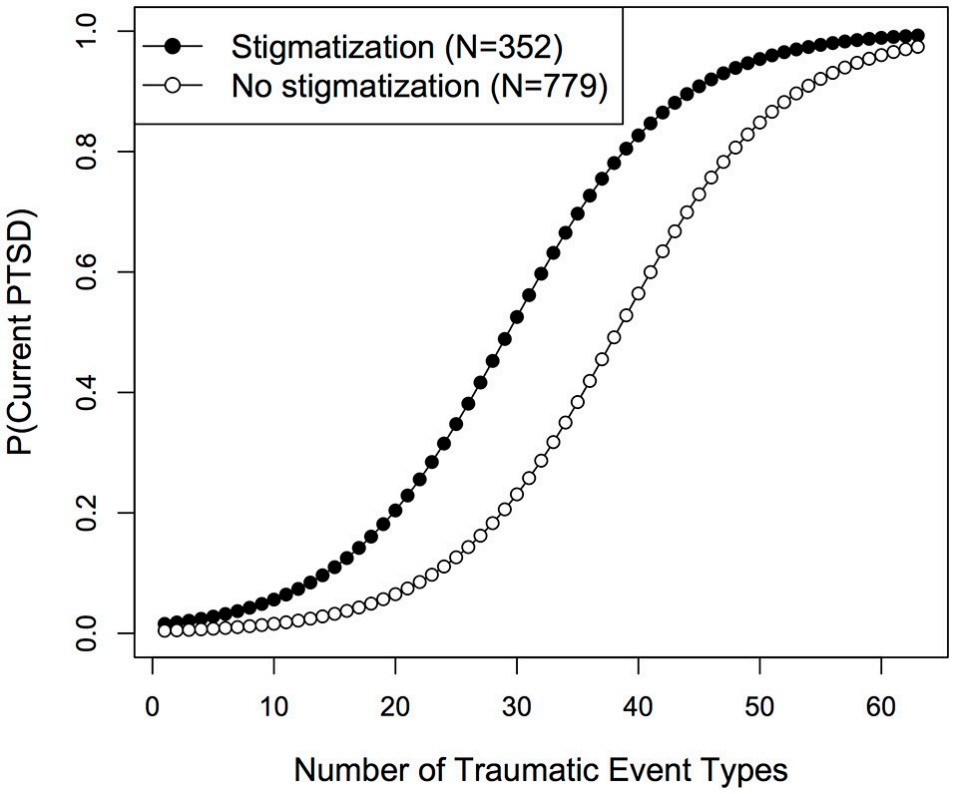
Fitted probabilities for current PTSD development among stigmatized and non-stigmatized individuals.

To further investigate whether stigmatization was associated with lifetime and current PTSD risk beyond other factors, i.e., trauma load, sex and age, logistic regression models were compared based on AIC. For both outcome variables, lifetime and current PTSD diagnosis, the main effect model including trauma load, stigmatization and sex revealed best model fit (see Supplementary Tables 1, 2). Neither the inclusion of additional variables nor the interaction trauma load × stigmatization improved model fit. For lifetime PTSD, the predictors trauma load [*LR*_(1)_ = 158.62, *p* < 0.001, OR (95% CI) = 1.12 (1.10–1.14)] and stigmatization [*LR*_(1)_ = 15.84, *p* < 0.001, OR (95% CI) = 2.21 (1.47–3.31)] reached statistical significance (Figure 1), while a marginally significant effect of sex [*LR*_(1)_ = 3.76, *p* = 0.052, OR (95% CI) = 0.74 (0.55–1.00)] was found. Similar results were observed for current PTSD and are displayed in Figure 2 [trauma load: *LR*_(1)_ = 259.64, *p* < 0.001, OR (95% CI) = 1.16 (1.14–1.19); stigmatization: *LR*_(1)_ = 61.47, p < 0.001, OR (95% CI) = 3.60 (2.61–4.97); sex: *LR*_(1)_ = 3.00, *p* = 0.083, OR (95% CI) = 0.76 (0.55–1.04)].

### Stigmatization and the likelihood of spontaneous remission

The frequency of spontaneous remission among stigmatized and non-stigmatized individuals can be obtained from Table 2. Of the *N* = 839 individuals fulfilling the criteria of a lifetime PTSD diagnosis, more than half (*N* = 466; 55.54%) had remitted spontaneously (note that the LRA had no more been active in Uganda in the recent past). Yet, *Chi*^2^-tests showed that among stigmatized individuals, spontaneous remission was less likely compared to non-stigmatized individuals [χ^2^(1) = 121.94, *p* < 0.001].

**Figure 3:**
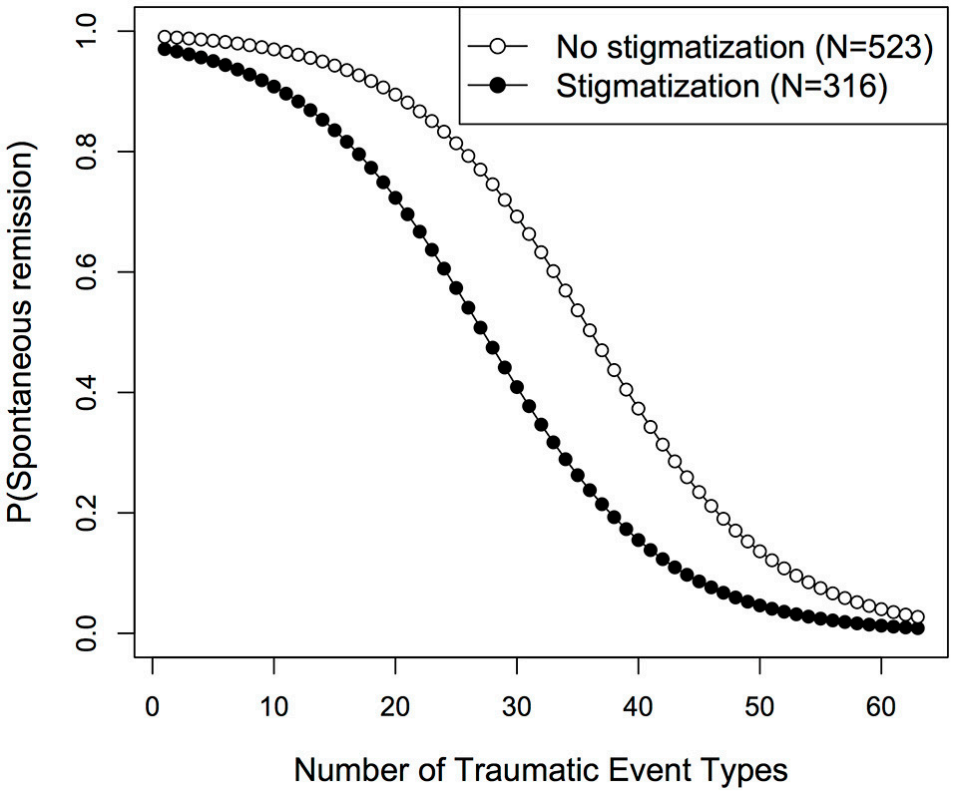
Fitted remission rates for stigmatized and non-stigmatized individuals.

Subsequently, logistic regression models with spontaneous remission as outcome variable were compared with the AIC. The best fit was obtained for a model including trauma load and stigmatization as predictors (Supplementary Table 3). We found significant main effects for both variables [trauma load: *LR*_(1)_ = 168.52, *p* < 0.001, OR (95% CI) = 0.88 (0.86–0.90); stigmatization: *LR*_(1)_ = 46.70, *p* < 0.001, OR (95% CI) = 0.31 (0.22–0.43)]. As shown in Figure 3, the predicted likelihood of spontaneous remission decreased with increasing trauma load in both groups. However, in the stigmatized group the probability for spontaneous remission was consistently lower than in the non-stigmatized group over all levels of trauma exposure.

### Stigmatization and therapy outcome

**Figure 4:**
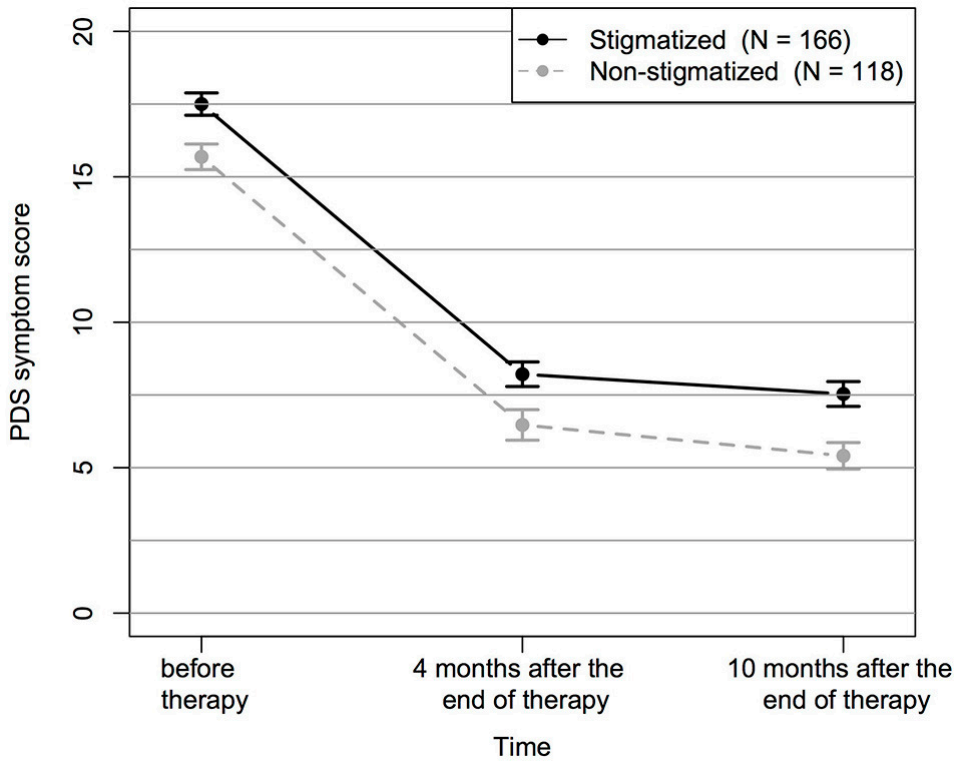
Change in PDS symptom score over time separately displayed for stigmatized and non-stigmatized individuals. Displayed are mean values and standard errors of the mean.

Finally, we investigated whether treatment outcome, i.e., symptom change in the PDS sum score over time, was predicted by stigmatization. Therefore, linear mixed effect models were compared (Supplementary Table 4) with regard to the AIC and revealed the best fit for the main effect model including time, trauma load, stigmatization, age and sex. Adding the interaction time × stigmatization did not improve model fit. As model residuals were non-normally distributed, statistical significance was confirmed by means of permutation tests (*p*_emp_). As displayed in Figure 4, we found significant main effects for the factor time [*F*_(2, 555)_ = 397.38, *p* < 0.001, p_emp_ < 0.001] and for stigmatization [*F*_(1, 279)_ = 5.57, *p* = 0.019, p_emp_ = 0.003]. Furthermore, trauma load [*F*_(1, 279)_ = 29.92, *p* < 0.001, p_emp_ < 0.001] and sex [*F*_(1, 279)_ = 9.25, p_emp_ = 0.003, p_emp_ = 0.006] significantly predicted treatment outcome, whereas a marginally significant effect was found for age [*F*_(1, 279)_ = 4.93, *p* = 0.027, p_emp_ = 0.073]. Treatment effect sizes for the stigmatized and non-stigmatized group can be obtained from Supplementary Table 5.

Additionally, we conducted *Chi*^2^-tests and observed a higher prevalence of a current PTSD diagnosis four and 10 months after the end of treatment in stigmatized as opposed to non-stigmatized individuals (Table 3). However, it is important to note that stigmatized individuals already presented with higher trauma load and a higher PTSD symptom severity before treatment (Table 4).

**Table 3:** Number of current PTSD cases and controls among the stigmatized and non-stigmatized group before therapy, four months after therapy and ten months after therapy.

	**Current PTSD diagnosis**	**No current PTSD diagnosis**	**Statistics^a^**
**Before therapy (t1)**			
*N* Stigmatization	166	0	n.a.
*N* No stigmatization	118	0	
**Four months after therapy (t2)**			
*N* Stigmatization	60	104	χ^2^(1) = 5.97, *p* = 0.015
*N* No stigmatization	26	91	
**Ten months after therapy (t3)**			
*N* Stigmatization	46	115	χ^2^(1) = 8.52, *p* = 0.004
*N* No stigmatization	15	100	

*PTSD, Posttraumatic Stress Disorder*.

a *Chi^2^-test for categorical data*.

**Table 4:** Comparison of demographic and clinical data of stigmatized and non-stigmatized individuals in the therapy group.

**Variable**	**Stigmatized group (*N* = 166)**	**Non-stigmatized group (*N* = 118)**	**Statistics**
*N* female (%)	97 (58.43)	63 (53.39)	Fisher's exact test: *p* = 0.467^a^
Age t1	Mdn = 30, IQR = 11	Mdn = 32, IQR = 16.75	*U* = 10978, *p* = 0.082^b^
Trauma load t1	M = 39.09; SD = 7.07	M = 34.54; SD = 6.93	*t* = −5.41, *p* < 0.001^c^
PDS sum score t1	Mdn = 17, IQR = 7	Mdn = 14, IQR = 7	*U* = 7540, *p* < 0.001^b^

*Mdn, Median; IQR, Interquartile range; M, Mean; SD, Standard deviation; PDS, Posttraumatic Stress Diagnostic Scale*.

a *Fisher's exact test for count data*.

b *Mann-Whitney U-test for continuous data, as model residuals were not normally distributed*.

c *Student's t-test for continuous data with normally distributed model residuals*.

To account for a potential effect of the time since the worst traumatic event on our outcome variables we repeated all our analyses additionally including this variable. For none of the outcome measures (i.e., PTSD lifetime and current diagnosis, remission, and therapy response) did the inclusion of the variable time since the worst traumatic event improve model fit according to AIC [(41); see also Supplementary Table 6). Furthermore, the results regarding the effects of traumatic load and stigmatization on all outcome measurements remained the same. Only minor changes with regard to the covariates age and sex were observed (Supplementary Tables 7, 8).

## Discussion

### Impact of stigma on trauma-related mental health

This study found a high prevalence of stigmatization in a sample of survivors of the LRA war in Northern Uganda. Similar to findings by Betancourt et al. (5), the main reasons for stigmatization in our sample were related to the individual's abduction by the LRA and the experiences made during the time in captivity, followed by HIV/AIDS and mental health problems. Even though the Ugandan government granted amnesty to the population of Northern Uganda (26), many former abductees face strong stigmatization by their families and communities. Considering the immense and long-lasting effects that the LRA war had on the Northern Ugandan community it is comprehensible that prejudice against people abducted by the LRA still exist. Even though the majority of the former LRA rebels were abducted forcefully, often as children, in the eyes of the community they represent a constant, unwanted reminder of this horrible time, the associated fear, anger and painful memories (25). Furthermore, society often holds a victim at least partially responsible for the experienced violence (49, 50). Additionally, a diagnosis of HIV/AIDS was a main reason for stigmatization. Fear of contamination [(51), for a review see (52)] and the lack of knowledge about disease transmission, development, and treatment options may contribute to the high prevalence of stigmatization among HIV positive individuals in this sample. It is further noteworthy that many survivors were stigmatized due to their mental health problems. Due to the lack of knowledge and superstition, the survivor's symptoms, e.g., flashbacks and dissociation, can be interpreted wrongly by the family and community. The belief in spirit possessions is highly prevalent in the Northern Ugandan culture, so that PTSD symptoms and the accompanying dysfunctionality may be misjudged (53, 54).

As previously shown, higher trauma load was associated with increased PTSD risk, and less spontaneous remission and therapy success [e.g., (19), Schneider et al., submitted]. However, we found that stigmatization is strongly associated with a higher prevalence of current and lifetime PTSD, a decreased likelihood of spontaneous remission and lower therapy success beyond the well-known effect of trauma load. In more detail, we observed strong main effects of stigmatization on lifetime and current PTSD. While the probability to suffer from PTSD increased with accumulating trauma load in both groups, stigmatized individuals had a higher PTSD prevalence across all levels of trauma exposure.

While the probability of spontaneous remission decreased in both groups with increasing trauma load, stigmatized individuals generally showed a reduced frequency of spontaneous remission. These results demonstrate the powerful influence that stigmatization has on mental health recovery. In addition, our analyses on treatment outcome point in a similar direction. While the treatment effect was similar in both groups, stigmatized individuals showed higher symptom scores before and after treatment. Accordingly, our results show that it is of utmost importance to address stigmatization in order to regain mental health.

Next to trauma load and stigmatization, gender was an important factor influencing PTSD symptom severity, with women indicating higher symptom scores than men. Furthermore, we found that the higher symptoms of women persisted throughout therapy, at the end of which women still presented with higher symptoms than men (55–57).

We also tested for a potential influence of the time interval between the worst traumatic experience and the interview on our results. However, the inclusion of this variable neither improved model fit, nor did it change the observed associations between stigmatization and all outcome measures. We can therefore conclude that our results are not biased by different time intervals since the worst traumatic event exposure.

Our results are in line with previous findings proposing general negative effects of stigmatization on mental health (4), as well as with studies showing that stigmatization has a strong impact on the severity of trauma-related symptoms, including PTSD (10, 58–61). However, it is important to note that the causality of the effects cannot be determined by cross-sectional studies. It is likely that both stigmatization and PTSD symptoms mutually maintain each other and should both be addressed in therapeutic interventions.

Based on the social stress theory (62), which considers stress and resources to act as mediators between social structure and poor health outcomes, Link and Phelan (3) proposed that the exposure to constant negative experiences through stigmatization predominantly leads to negative health outcomes and social adjustment difficulties. Thus, stigmatization might diminish the individuals' access to resources and coping strategies (62). Based on this theory, one might assume the lack of resources due to the prevalent stigmatization and discrimination as a possible reason for the increased PTSD prevalence, the decreased rates of spontaneous remission and the lower therapy success found in this study.

Our results point out the importance of interventions which not only focus on individual PTSD treatment, but also include family-oriented approaches and community interventions to reduce stigmatization. In particular, the successful reintegration of former child soldiers appears to be highly dependent on family and community acceptance (8, 9). Therefore, awareness-raising interventions are required and should enhance the knowledge of the community about the traumatic character of the returnees' experiences in the LRA, the potentially resulting mental health impairments, and the negative impact of stigmatization.

### Strengths and limitations

A strength of this study is the exceptionally large sample used for investigations on PTSD risk, and spontaneous remission as well as the longitudinal design of the therapy analyses. Stigmatization was further investigated for many different outcomes, and trauma load was assessed and included in all analyses, as were other predisposing factors such as sex and age. All diagnostic interviews were standardized, and the therapy group received manualized treatment.

This study investigated stigmatization as a possible additional risk factor for psychopathology beyond the influence of cumulative trauma in rebel war survivors from Northern Uganda. However, given the unique research context, the limitation should be noted that no validated instrument to assess stigmatization has been available for the current context. Consequently, we were neither able to differentiate various forms of stigmatization, nor to assess and include whether the participant was stigmatized by the community or family, which might be an important factor. A qualitative analysis of participants' responses indicated LRA-related stigmatization as the predominant cause of stigmatization-related social rejection. However, future studies might aim to develop psychometric measures that allow for the separate assessment of discrimination and stigmatization as well as stigmatization sub-forms, e.g., society and self/internalizing stigmatization (51), in conflict survivors. Finally, we have to note that stigmatization and traumatic event exposure were correlated, which makes it difficult to distinguish their specific effects on psychopathology.

### Conclusions and future directions

In summary, this study provides strong evidence that the experience of stigmatization and discrimination combined with trauma load is associated with PTSD prevalence, the likelihood of spontaneous remission and therapy success. Therefore, it will be helpful to develop programs that sensitize the population regarding negative impacts of stigmatization, and to highlight the necessity to reduce stigmatization and discrimination in order to allow survivors to recover and reintegrate into the community. Furthermore, it is important to consider the handling of stigmatization experiences as an additional treatment target besides PTSD symptom reduction. Previous findings in the present sample showed that approximately 20% of the PTSD patients do not profit from trauma therapy to the extent that the diagnostic criteria of PTSD are no longer fulfilled (Schneider et al., submitted). Therefore, interventions targeting stigmatization and including the family and community might lead to better therapy outcome and long-term therapy success.

## Data availability statement

The datasets analyzed in this manuscript are not publicly available due to data protection of participants as the datasets contain sensitive clinical information. Requests to access the data can be directed to iris.kolassa@uni-ulm.de.

## Author contributions

SW and I-TK developed the study concept. AS, SW, and AP conducted the study setup and data collection under supervision of I-TK and TE. AS and DC performed the statistical data analysis and drafted the paper under supervision of SW. All authors critically revised and approved the final version of the manuscript for submission.

### Conflict of interest statement

The authors declare that the research was conducted in the absence of any commercial or financial relationships that could be construed as a potential conflict of interest.
