# Supplementary material for: Stigmatization Is Associated With Increased PTSD Risk After Traumatic Stress and Diminished Likelihood of Spontaneous Remission–A Study With East-African Conflict Survivors
Source: Front Psychiatry. 2018 Oct 10;9:423. doi: 10.3389/fpsyt.2018.00423 (PMC6191513; doi:10.3389/fpsyt.2018.00423)
Supplement: Supplementary file 1 [file Data_Sheet_1.docx]

Supplementary Material

**Stigmatization Is Associated with Increased PTSD Risk After Traumatic Stress and Diminished Likelihood of Spontaneous Remission – A Study with East-African Conflict Survivors**

**Anna Schneider^*^, Daniela Conrad^*^, Anett Pfeiffer, Thomas Elbert, Iris-Tatjana Kolassa, Sarah Wilker**

*** Correspondence:**

Anna Schneider, anna.schneider@uni-ulm.de,

Daniela Conrad, daniela.conrad@uni-konstanz.de and

Sarah Wilker, sarah.wilker@uni-bielefeld.de

**Supplementary Methods**

# *Further information on the drop-out of individuals from the therapy sample*

Of the *N* = 29 individuals who dropped-out of the study, *N* = 7 did not complete the treatment, *N* = 3 moved away, *N* = 5 could not be found for both follow-up assessments, *N* = 1 could not be interviewed for both follow-ups due to a stroke, *N* = 1 person died after the end of therapy due to mob justice, and *N* = 1 was an extremely severe case, where an international expert took over the treatment, *N* = 10 participants were excluded due to signs of current alcohol abuse and *N* = 1 participant took unknown psychiatric medication at the beginning of the therapy.

# Supplementary Tables

Supplementary Table 1. *Model comparison for analyses on lifetime PTSD diagnosis*

| **Model** | **AIC** |
| --- | --- |
| Lifetime PTSD diagnosis ~ trauma load | 1084.87 |
| Lifetime PTSD diagnosis ~ trauma load + stigmatization | 1069.49 |
| Lifetime PTSD diagnosis ~ trauma load × stigmatization | 1069.80 |
| **Lifetime PTSD diagnosis ~ trauma load + stigmatization + sex** | **1067.82** |
| Lifetime PTSD diagnosis ~ trauma load + stigmatization + sex + age | 1068.99 |

*Note.* The best fitting model is marked bold. AIC comparisons are based on *N* = 1130 (one individual was excluded from the dataset for missing age).

Supplementary Table 2. *Model comparison for analyses on current PTSD diagnosis*

| **Model** | **AIC** |
| --- | --- |
| Current PTSD diagnosis ~ trauma load | 1056.54 |
| Current PTSD diagnosis ~ trauma load + stigmatization | 994.28 |
| Current PTSD diagnosis ~ trauma load × stigmatization | 995.11 |
| **Current** **PTSD diagnosis ~ trauma load + stigmatization + sex** | **993.28** |
| Current PTSD diagnosis ~ trauma load + stigmatization + sex + age | 993.14 |

*Note.* The best fitting model is marked bold. AIC comparisons are based on *N* = 1130 (one individual was excluded from the dataset for missing age).

Supplementary Table 3. *Model comparison for analyses on spontaneous remission*

| **Model** | **AIC** |
| --- | --- |
| Spontaneous remission ~ trauma load | 909.22 |
| **Spontaneous remission** **~ trauma load + stigmatization** | **864.53** |
| Spontaneous remission ~ trauma load × stigmatization | 864.88 |
| Spontaneous remission ~ trauma load + stigmatization + sex | 864.11 |
| Spontaneous remission ~ trauma load + stigmatization + sex + age | 865.31 |

*Note.* The best fitting model is marked bold. AIC comparisons are based on *N* = 839 (no individuals had to be excluded for missing data).

Supplementary Table 4. *Model comparison for analyses on treatment outcome*

| **Model** | **AIC** |
| --- | --- |
| PDS sum score ~ time + stigmatization | 5126.90 |
| PDS sum score ~ time × stigmatization | 5130.63 |
| PDS sum score ~ time + stigmatization + trauma load | 5102.31 |
| PDS sum score ~ time + stigmatization + trauma load + sex | 5095.31 |
| **PDS sum score ~ time + stigmatization + trauma load + sex + age** | **5092.40** |

*Note.* The best fitting model is marked bold. AIC comparisons are based on *N* = 284 (no individuals had to be excluded for missing data).

Supplementary Table 5.

*Mean and standard deviation of PDS scores before therapy (t1), 4-months after therapy (t2) and at 10-months follow up (t3).*

|  | **Stigmatization**  **yes/no** | **Mean (s.d.) PDS score** | | | **Change Score**  **t1 - t2**^c^ | **Effect size Cohen’s D**  **t1 - t2**^d^ | **Change Score**  **t1 - t3**^c^ | **Effect size Cohen’s D**  **t1 - t3**^d^ | **Change Score**  **t2 - t3**^c^ | **Effect size Cohen’s D**  **t2 - t3**^d^ |
| --- | --- | --- | --- | --- | --- | --- | --- | --- | --- | --- |
|  |  |  | | |  |  |  |  |  |  |
|  |  | **t1** | **t2**^a^ | **t3**^b^ |  |  |  |  |  |  |
|  | Stigmatized  (*N* = 166) | 17.50 (4.95) | 8.21 (5.41) | 7.53  (5.41) | - 9.24  (5.96) | 1.79 | - 9.93  (6.95) | 1.92 | - 0.69  (6.43) | 0.13 |
|  | Non-stigmatized  (*N* = 118) | 15.69 (4.78) | 6.47 (5.67) | 5.41  (4.88) | - 9.72  (6.79) | 1.76 | - 10.19  (6.00) | 2.13 | - 1.18 (6.32) | 0.20 |

*Note:* PDS = Posttraumatic Stress Diagnostic Scale

^a^ Three individuals were not found for the post-test 4-months after therapy.

^b^ Eight individuals were not found for 10-months follow-up.

^c^ Change score describes the averaged difference in within-group PDS sum scores between pre-treatment and 4-months follow up, pre-treatment and 10-months follow-up, and between 4- and 10-months follow up, respectively.

^d^ Cohen’s *D* describes the treatment effect size between pre-treatment and 4-months follow up, pre-treatment and 10-months follow-up, and between 4- and 10-months follow up assessment calculated separately for stigmatized and non-stigmatized.

Supplementary Table 6. *Model comparison for all analyses excluding and including the time since worst traumatic event-variable*

| **Outcome Variable** | **Predictors** | **AIC** |
| --- | --- | --- |
| Lifetime PTSD^1^ | Trauma load + stigmatization + sex | 1060.38 |
| Lifetime PTSD^1^ | Trauma load + stigmatization + sex + time since worst event | 1062.24 |
| Current PTSD^1^ | Trauma load + stigmatization + sex | 992.57 |
| Current PTSD^1^ | Trauma load + stigmatization + sex + time since worst event | 994.08 |
| Remission^2^ | Trauma load + stigmatization | 864.53 |
| Remission^2^ | Trauma load + stigmatization + time since worst event | 866.28 |
| Therapy Outcome^3^ | Trauma load + stigmatization + sex + age | 5076.10 |
| Therapy Outcome^3^ | Trauma load + stigmatization + sex + age + time since worst event | 5077.11 |

*Note:* The sample used for analyses on lifetime and current PTSD diagnosis included *N* = 1131 individuals. For AIC comparisons individuals with missing data in any of the variables included into the model were excluded.

^1^ *N* = 1126 (5 of 1131 individuals were excluded for missing data in the time since worst event variable)

^2^ *N* = 839 (no individuals had to be excluded for missing data)

^3^ *N* = 283 (one individual was excluded for missing data in the time since worst event variable)

AIC = Akaike’s information criteria; PTSD = Posttraumatic Stress Disorder; PDS = Posttraumatic Stress Diagnostic Scale

Supplementary Table 7. *Results of likelihood ratio tests for lifetime and current PTSD diagnosis and remission including the time since worst traumatic event-variable*

| **Outcome Variable** | **Predictors** | **Statistic** | ***p*-value** | **OR (95%-CI)** |
| --- | --- | --- | --- | --- |
| Lifetime PTSD | Trauma load | *LR*(1) = 149.71 | *p* < .001 | 1.12 (1.10-1.14) |
|  | Stigmatization | *LR*(1) = 15.24 | *p* < .001 | 2.18 (1.45-3.27) |
|  | Sex | *LR*(1) = 4.28 | *p* = .039 | 0.73 (0.53-0.98) |
|  | Time since worst event | *LR*(1) = 0.14 | *p* = .712 | 1.01 (0.98-1.03) |
| Current PTSD | Trauma load | *LR*(1) = 255.22 | *p* < .001 | 1.16 (1.14-1.19) |
|  | Stigmatization | *LR*(1) = 60.03 | *p* < .001 | 3.56 (2.58-4.92) |
|  | Sex | *LR*(1) = 3.09 | *p* = .079 | 0.75 (0.55-1.03) |
|  | Time since worst event | *LR*(1) = 0.49 | *p* = .485 | 0.99 (0.96-1.02) |
| Remission | Trauma load | *LR*(1) = 167.704 | <.001 | 0.88 (0.86-0.90) |
|  | Stigmatization | *LR*(1) = 46.10 | <.001 | 0.31 (0.22-0.44) |
|  | Time since worst event | *LR*(1) = 0.24 | .623 | 1.01 (0.98-1.04) |

*Note: LR =* Likelihood Ratio Test; OR = odds ratio; 95%-CI = 95% Confidence Interval; PTSD = Posttraumatic Stress Disorder

Supplementary Table 8. *Results of linear mixed effect model calculated to investigate the effect of stigmatization on therapy outcome including the time since worst traumatic event-variable*

| **Outcome Variable** | **Predictors** | **Statistic** | ***p*-value** | ***p*_emp_** |
| --- | --- | --- | --- | --- |
| Therapy outcome | Time | F_2,553_ = 395.82 | *p* < .001 | *p*_emp_ < .001 |
|  | Trauma load | F_1,277_ = 29.20 | *p* < .001 | *p*_emp_ < .001 |
|  | Stigmatization | F_1,277_ = 5.47 | *p* = .020 | *p*_emp_ = .016 |
|  | Sex | F_1,277_ = 9.64 | *p* = .002 | *p*_emp_ = .003 |
|  | Age | F_1,277_ = 2.12 | *p* = .147 | *p*_emp_ = .150 |
|  | Time since worst event | F_1,277_ = 0.99 | *p* = .322 | *p*_emp_ = .317 |

Note: *p*_emp_ = Empirical p-value based on 1,000 permutations
